# Long-term prognostic impact of one-year change in left ventricular function after a myocardial infarction: insights from the REBUS cohort

**DOI:** 10.1093/ehjimp/qyag005

**Published:** 2026-01-12

**Authors:** Joel Lenell, Christina Christersson, Bertil Lindahl, Jonas Oldgren, Frank A Flachskampf, Tomasz Baron

**Affiliations:** Department of Medical Sciences, Cardiology, Uppsala University, Sjukhusvägen 7, 753 09 Uppsala, Sweden; Uppsala Clinical Research Center, Uppsala University, Dag Hammarskjölds Väg 38, 751 85 Uppsala, Sweden; Department of Medical Sciences, Cardiology, Uppsala University, Sjukhusvägen 7, 753 09 Uppsala, Sweden; Uppsala Clinical Research Center, Uppsala University, Dag Hammarskjölds Väg 38, 751 85 Uppsala, Sweden; Department of Medical Sciences, Cardiology, Uppsala University, Sjukhusvägen 7, 753 09 Uppsala, Sweden; Uppsala Clinical Research Center, Uppsala University, Dag Hammarskjölds Väg 38, 751 85 Uppsala, Sweden; Department of Medical Sciences, Cardiology, Uppsala University, Sjukhusvägen 7, 753 09 Uppsala, Sweden; Uppsala Clinical Research Center, Uppsala University, Dag Hammarskjölds Väg 38, 751 85 Uppsala, Sweden; Department of Medical Sciences, Cardiology, Uppsala University, Sjukhusvägen 7, 753 09 Uppsala, Sweden; Department of Clinical Physiology and Cardiology, Uppsala University, Uppsala, Sweden; Department of Medical Sciences, Cardiology, Uppsala University, Sjukhusvägen 7, 753 09 Uppsala, Sweden; Uppsala Clinical Research Center, Uppsala University, Dag Hammarskjölds Väg 38, 751 85 Uppsala, Sweden

**Keywords:** global longitudinal strain, left ventricular ejection fraction, outcome, prognosis

## Abstract

**Aims:**

Acute myocardial infarction (MI) management has changed in the last decades with the introduction of routine early invasive revascularization and potent antiplatelet therapy followed by an improvement in patient prognosis. This study aimed to evaluate if it remains of long-term prognostic value to repeat the assessment of systolic function, determining change in ejection fraction (LVEF) and global longitudinal strain (GLS), within a year after an MI.

**Methods and results:**

Patients hospitalized with acute MI (*n* = 256) were recruited in 2010 through 2012 at Uppsala University Hospital. All participants underwent an echocardiographic examination during the index hospital stay and at 1-year following discharge. Outcome data of time to first heart failure hospitalization or all-cause death was collected up until July 2022. Mean age was 66 years (80% men). Median follow-up was 11.7 (IQR 10.9–12.4) years with 63 observed events. A 1-year improvement in LVEF and GLS was not associated with the outcome [LVEF: adjusted HR 1.03 (CI 95% 0.51–2.05); GLS: adjusted HR 1.05 (CI 95% 0.55–2.04)], whereas a deterioration in GLS was associated with a higher event rate [adjusted HR 5.60 (CI 95% 2.04–15.39)]. Baseline GLS offered a higher C-index than LVEF [0.71 (0.65–0.78) vs. 0.68 (0.61–0.75)].

**Conclusion:**

Improved systolic function after MI did not add incremental long-term prognostic information beyond the baseline systolic function, however, a deterioration in GLS may be associated with worse long-term prognosis. Our results favor a clinical strategy of risk stratification based on GLS rather than LVEF to enhance clinical risk prediction.

## Graphical Abstract

**Graphical Abstract:**
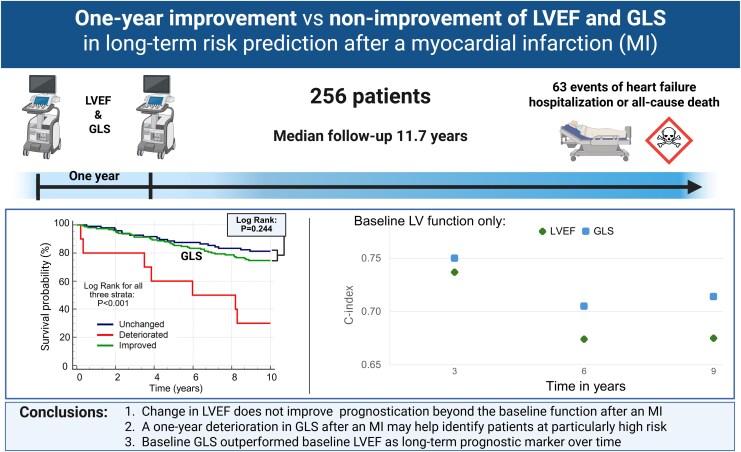

## Introduction

In the current era of early percutaneous coronary intervention (PCI) and dual anti-platelet therapy in myocardial infarction (MI) management, most patients survive with normal or near-normal left ventricular function.^1^ Echocardiographic assessment of left ventricular ejection fraction (LVEF) is a well-established measurement of systolic left ventricular function dating back to 1974 and much of the literature in support of LVEF as a management directing metric following MI was published before adoption of today’s guideline endorsed treatments.^2,3^ Consequently, there is a need for continued evaluation of traditional and new metrics of risk as clinical management changes over time.

Recent large randomized controlled trials of new heart failure treatments, such as Sacubitril-Valsartan and Sodium-glucose cotransporter 2 (SGLT-2)-inhibitors, have failed to improve life expectancy and reduce heart failure (HF) hospitalization compared to placebo in patients with MI.^4,5^ Traditional beta-blockers have also failed to prevent death or recurrent MI in a recent trial.^6^

Given that previous studies in patient populations recruited before widespread adoption of early invasive revascularization and potent anti-thrombotic treatment reported better prognosis among individuals with improved LVEF over time after MI; it is of interest to evaluate the importance of change in LV function among modern-day MI patients.^7^ In the subacute setting of MI, there is myocardial dysfunction due to irreversible necrosis but also due to ischaemia-related reversible stunning.^8^ Days to weeks following an MI, systolic function often improve as myocardial stunning spontaneously resolve and whether this improvement translates into an altered prognosis is not well studied.

Global longitudinal strain (GLS), a metric of systolic left ventricular function, has repeatedly been suggested to offer prognostic information in MI. Although being recognized to provide better prognostic guidance than LVEF in a wide range of cardiac diseases,^9^ GLS was recently reported not to significantly improve long-term discrimination of high-risk individuals following MI beyond what LVEF could provide in a real-world clinical setting.^10^

In this study, we hypothesized that patients with a 1-year improvement in systolic function would have a lower risk of long-term HF hospitalization or death than those with no improvement after an MI. Conversely, we assumed that those with a reduction in systolic function would present an increased risk of this outcome. Hence, this study effectively evaluates the prognostic benefit of repeated assessments of systolic function in a real-world MI population.

## Methods

### Study population

The RElevance of Biomarkers for future risk of thromboembolic events in UnSelected post-myocardial infarction patients (REBUS) cohort was recruited in 2010 through 2012 at Uppsala University Hospital in Sweden (NCT01102933, ClinicalTrials.gov). In REBUS, 421 patients with MI were enrolled (see Supplementary data online, Supplemental Material page 1: Inclusion and exclusion criteria) for a repeat echocardiographic exam, 1 year after their MI, and followed longitudinally to identify predictors of adverse outcomes. The overall population characteristics has previously been described elsewhere.^11^

For the purpose of this study, patients with available echocardiographic exams from both the index hospital stay, and from the 1-year follow-up, were included. Participants who died within a year from the baseline hospitalization, before the follow-up scan, and those scanned with degraded image quality, preventing delineation of at least two neighbouring myocardial segments in either scan, were excluded. All patients were treated according to clinical routine based on current international management guidelines at the time of enrollment. This involved primary percutaneous coronary intervention (PCI) in patients suffering an ST-elevation myocardial infarction (STEMI) and early revascularization, typically within 48 h of admission, in those suffering a non-ST-elevation myocardial infarction (NSTEMI). Data on medical history and previous treatments were collected before discharge.

**Figure 1:**
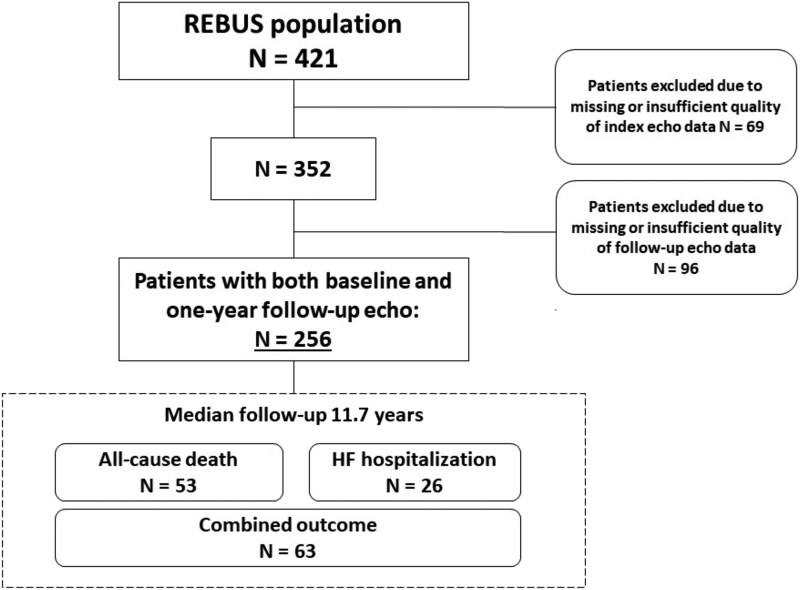
Inclusion chart.

Of the 421 patients enrolled, 256 were included with available echocardiographic data from both scans. Among excluded patients, nine died within the first year of discharge and another 18 withdrew consent. Forty-three exams were excluded due to insufficient image quality while the remainder of excluded subjects did not have their images available in the local database (*Figure 1*).

### Outcomes

The primary outcome was time to first HF hospitalization or all-cause death measured from the second scan, 1 year after enrolment. Patient outcome data were collected up until July 2022. Mortality data were retrieved from the National Cause of Death registry and time to first HF hospitalization was retrieved from the Swedish Patient Registry (PAR). HF hospitalization was defined as the first hospital admission with heart failure as the primary discharge diagnosis, coded according to one of the following ICD codes: I50.0, I50.1, or I50.9.

### Echocardiography

Baseline echocardiographic exams were performed within 72 h of admission and a follow-up exam was performed at 12 months following discharge. All exams were analysed using the software TomTec-Arena version 2.50 (TomTec, Unterschleißheim, Germany). Peak endocardial GLS acquisitions from the apical 4-, 2-, and 3-chamber views were software automated using speckle tracking with manual corrections applied in case of suboptimal endocardial tracings. No segmental data were assessed and no information on valvular heart disease was retrieved. Biplane LVEF was calculated according to Simpson’s method of discs. All manual corrections and decisions on exclusion were done by one single experienced imager (first author).

Significant improvement or deterioration in LVEF and GLS was defined according to the previously reported smallest detectable change; represented by a relative change of at least 14.2% and 14.7%, respectively, assuming that changes beyond these relative thresholds represent a biological change in function, rather than methodological noise.^12^ Twelve included patients were in atrial fibrillation at either of the two exams in whom care was taken to register GLS at somewhat regular RR-intervals.

### Statistics

Normality was assessed by the Shapiro-Wilk test and visual inspection of frequency histograms. Variables of approximately normal distribution were reported as mean and standard deviation (SD) and skewed variables were reported as median and inter quartile range (IQR). Categorical variables were reported as frequency and percentage of the total population.

Baseline characteristics among individuals with no change, improvement or a reduction in LVEF were reported separately. Differences in continuous variables were tested by ANOVA with Bonferroni corrections and differences in categorical variables were tested with Chi-square statistics.

Both LVEF and GLS were categorized into three-strata variables of non-change, improvement or deterioration.^12^ Non-change in LVEF and GLS was defined as the interval of less than ±14.2% and ±14.7% in relative change for the respective metrics. Testing of inter-scan differences in LVEF and GLS was done with the Wilcoxon signed rank test.

Two unadjusted Kaplan–Meier curves stratified by direction of change in LVEF and GLS were presented to illustrate the prognosis over time per group. A table with event counts and hazard ratios for each stratum was also reported including both unadjusted analyses and analyses adjusted for the baseline systolic function to account for regression-to-the-mean.

Univariable Cox regression models were built to examine the association between patient characteristics and systolic function measurements with time to first HF hospitalization or all-cause death as dependent variable. Harrell’s C-index was assessed for each unadjusted variable to evaluate prognostic accuracy. Harrell’s C-index was furthermore determined for baseline LVEF and GLS with the outcome right-censored at 3, 6, and 9 years respectively to depict their individual discriminative value over time.

In a second step, multivariable Cox regression models were built to evaluate the association of change in LVEF and GLS to the combined outcome. Change in LVEF and GLS was studied in separate regression models due to their known covariation and these models were adjusted by variables that emerged with statistical significance in the univariable analyses alongside baseline systolic function by either LVEF or GLS, depending on which variable was studied, to account for regression-to-the-mean.^13^ Improvement and deterioration in the two echo measurements was tested against non-change in all multivariable models.

Additional explorative Cox regression analyses were performed examining absolute continuous changes in LVEF and GLS. Bootstrapped sensitivity analyses were also performed to evaluate the prognostic stability of deterioration in GLS as only a few patients presented with a deterioration (*n* = 10).

Extent of obstructive coronary artery disease and the impact of chosen invasive treatment strategy on LVEF was explored in sub-analyses using linear regression.

Statistical testing was performed in SPSS version 28.0.0.1 (IBM Corp. Released 2021. IBM SPSS Statistics for Windows, Version 28.0. Armonk, NY: IBM Corp) or in R (4.0.2). All hypotheses were two-sided and level of statistical significance was set to *P* < 0.05.

This study was approved by the local ethics committee in Uppsala (DNR 2009/210) and was carried out in accordance with the Helsinki declaration. All patients provided written informed consent on enrollment.

## Results

### Population characteristics

Two-hundred and fifty-six (256) patients with completed serial echocardiographic exams were included (mean age 66 years, 80% men) (*Figure 1*). Included patients were younger than excluded patients (66 years [SD ±10] vs. 69 years [SD ±10], *P* = 0.018) and more often suffered a STEMI (51.2% vs. 38.2%, *P* = 0.009). There were no statistically significant differences in sex or systolic function between included and excluded patients (see Supplementary data online, *Table S1*). Among the 9 patients who died before their second scan, mean age was 71 years (SD ±7) and 5 (55.6%) had a previous MI (see Supplementary data online, *Table S2*) compared to 17.2% among those included. Median follow-up was 11.7 years [inter quartile range (IQR): 10.9–12.4] measured from the second echocardiographic exam with 63 events of HF hospitalization or all-cause death.

PCI was performed in 231 patients (90.2%) and another 7 (2.7%) underwent coronary artery bypass surgery (CABG) (see Supplementary data online, *Table S3*). One vessel-disease was associated with higher baseline LVEF [beta coefficient: 2.64 (CI 95% −0.31 to 5.59)] and three-vessel disease was associated with worse baseline LVEF [beta coefficient −2.81 (CI 95% −7.86 to 1.76)] although these differences did not meet statistical significance (see Supplementary data online, *Table S4*). The chosen revascularization strategy (CABG, PCI, full revascularization and no revascularization) was not predictive of baseline LVEF.

Patients with improved LVEF more often suffered a STEMI than patients with unchanged or deteriorated LVEF and were less likely to have pre-existing hypertension (*Table 1*). Those with improved or deteriorated LVEF presented with higher Peak Troponin I and higher C-reactive protein than those with unchanged function. Otherwise, there were no statistically significant baseline differences between patients with unchanged, improved or reduced LVEF over 1 year. Participants with a subnormal LVEF (<50%) at the follow-up scan (*n* = 39) had a median baseline LVEF at 37% (IQR 30–41) and a median follow-up LVEF at 41% (IQR 34–45).

**Table 1:** Baseline characteristics

	All *n* = 256	Unchanged LVEF *n* = 141	Reduced LVEF *n* = 10	Improved LVEF *n* = 105
Male sex, *n* (%)	205 (80.1)	110 (78.0)	8 (80.0)	87 (82.9)
Age, mean (SD)	66 (10)	66 (10)	69 (9)	66 (10)
Diagnosis of MI				
STEMI, *n* (%)	131 (51.2)	63 (44.7)	5 (50.0)	63 (60.0)^a^
NSTEMI, *n* (%)	125 (48.8)	78 (55.3)	5 (50.0)	42 (40.0)^a^
Biomarkers, median (IQR)				
Peak Troponin I, µg/L	10.2 (2.1–47.7)	6.9 (1.9–25.7)	25.7 (4.7–53.0)	22.0 (4.0–50.0)^a^
Hemoglobin, g/L	141 (130–149)	140 (127–148)	139 (133–149)	141 (132–149)
Creatinine, µmol/L	83 (70–93)	82 (70–94)	85 (72–116)	83 (72–92)
hsCRP, mg/L	2.4 (1.2–5.2)	2.2 (1.1–4.2)	2.2 (1.9–6.5)	2.9 (1.6–10.0)^a^
Medical history before MI, *n* (%)				
Hypertension	130 (50.8)	80 (56.7)	9 (90.0)^a^	41 (39.0)^a^
Diabetes mellitus	36 (14.1)	17 (12.1)	2 (20.0)	17 (16.2)
Heart failure	13 (5.1)	6 (4.3)	1 (10.0)	6 (5.7)
Chronic kidney disease	5 (2.0)	3 (2.1)	0 (0.0)	2 (1.9)
History of myocardial infarction	44 (17.2)	25 (17.7)	2 (20.0)	17 (16.2)
History of stroke	9 (3.5)	4 (2.8)	1 (10.0)	4 (3.8)

Comparison of between-group characteristics by 1-year change in LVEF.

^a^Statistically significant difference compared to the combined two other categories.

**Figure 2:**
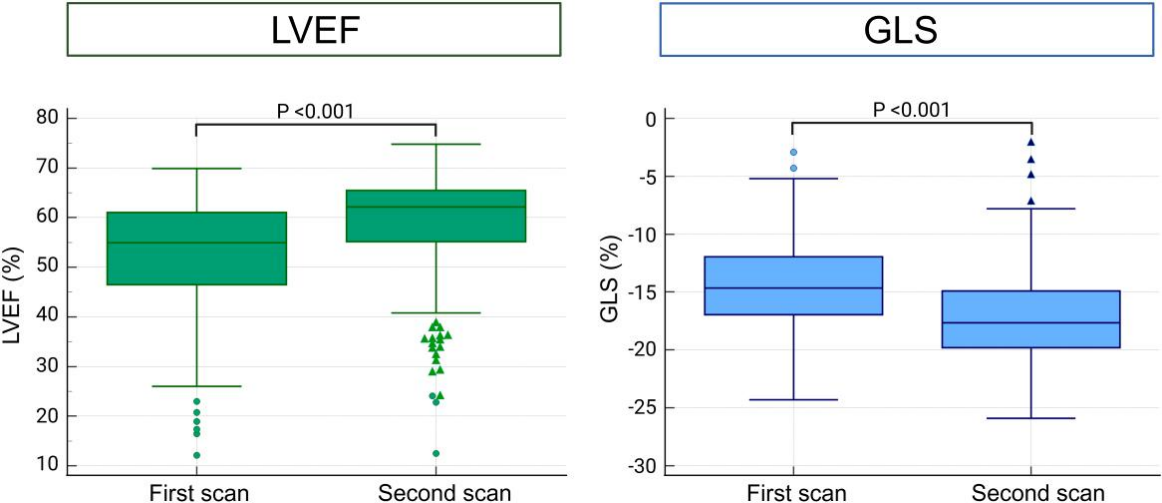
One year change in LVEF and GLS after MI. Description: Midline = median; box = inter-quartile range; whiskers = 1.5 × inter-quartile range; first-order outliers = > 1.5 × inter-quartile range; second-order outliers = > 3.0 × inter-quartile range.

At discharge after the index hospitalization, 83.2% were prescribed a renin-angiotensin-aldosterone system (RAAS)-inhibitor and 92.2% were prescribed a beta-blocker (*Table 2*). After 1 year, 77.7% were taking a RAAS-inhibitor and 84.8% were taking a beta-blocker. There was a net improvement in both LVEF [55% (46–61) vs. 62% (55–66), *P* < 0.001] and GLS [−14.7% (−17.0 to −11.9) vs. −17.7% (−19.8 to −14.9), *P* < 0.001] over 1 year in the overall population (*Figure 2*). Ten patients experienced a significant decrease in GLS and LVEF, respectively, whereas 150 patients had an increase in GLS and 105 had an increase in LVEF. A majority of patients (65%) with improved LVEF presented with subnormal baseline function [median LVEF 47% (39–53), median GLS −13.7% (−15.7 to −11.0)]. At 1 year, there was no statistically significant difference in LVEF between those with improved and those with unchanged LVEF [61% (55–66) vs. 63% (58–66), *P* = 0.293], whereas patients with improved GLS presented with better GLS at 1 year than those with no change [−18.2% (−20.4 to −15.4) vs. −17.1% (−18.9 to −14.7), *P* = 0.008].

**Table 2:** Treatment and echocardiographic measurements at discharge and after one year

	Index hospitalization	1-year follow-up
Treatments		
Aspirin, *n* (%)	253 (98.8)	225 (87.9)
P2Y12-inhibitor, *n* (%)	253 (98,8)	16 (6.0)
Oral anticoagulant, *n* (%)	12 (4.7)	26 (10.2)
Beta-blocker, *n* (%)	236 (92.2)	217 (84.8)
RAAS-inhibitor, *n* (%)	213 (83.2)	199 (77.7)
Statin, *n* (%)	246 (96.1)	212 (82.8)
Echocardiography		
LVEF (%), median (IQR)	55 (46–61)	62 (55–66)
LV GLS (%), median (IQR)	−14.7 (−17.0 to −11.9)	−17.7 (−19.8 to −14.9)
LA volume (ml), median (IQR)	62 (49–74)	62 (50–76)
LA reservoir strain (%), median (IQR)	27 (21–32)	29 (22–33)

Both patients suffering the combined outcome and those with no events demonstrated a net improvement in LVEF and GLS over 1 year although patients with events had relatively worse LVEF and GLS at both scans (see Supplementary data online, *Figure S1*). Twenty-eight patients with a normal baseline systolic function (LVEF ≥ 50%) met the combined endpoint, answering for 44% of total events.

### Outcome analysis

**Figure 3:**
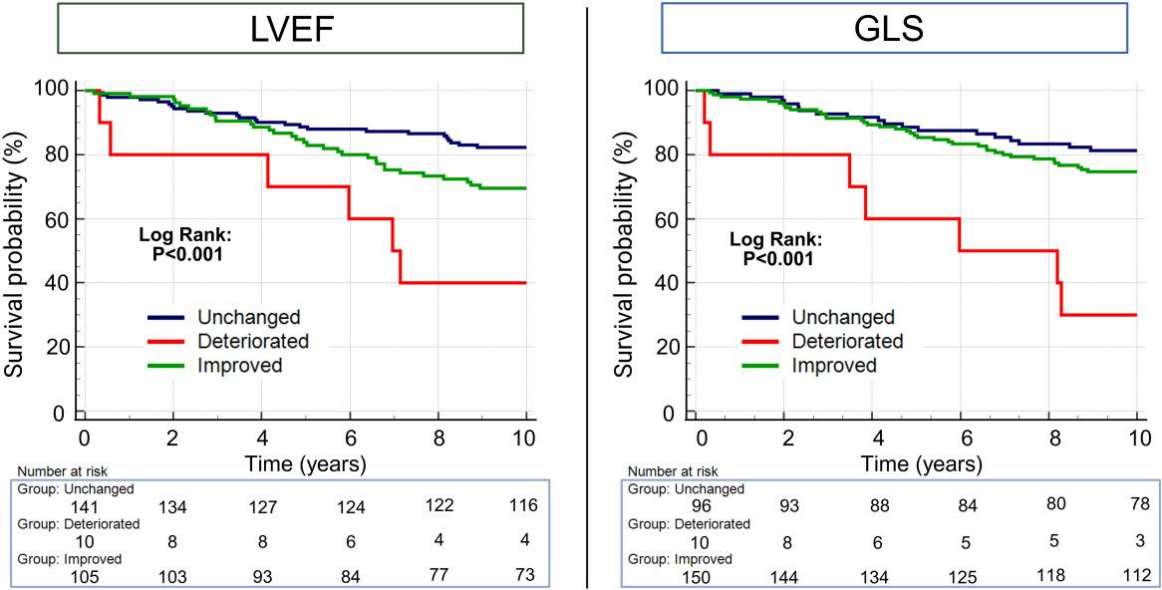
Kaplan–Meier curves with time to HF hospitalization or all-cause death from the second scan as dependent variable stratified by direction of change in LVEF (left) and GLS (right). Description: GLS—global longitudinal strain; LVEF—left ventricular ejection fraction.

The Kaplan–Meier curves depict that those with a 1-year reduction in LVEF had a 60% probability of the combined outcome during follow-up and those with a reduction in GLS had a 70% probability of the combined outcome (*Figure 3*). Patients with an improvement in systolic function demonstrated a higher event-rate than those with no change in this unadjusted analysis.

Cox regression models for the three strata in *Figure 3* (unchanged, deteriorated and improved) demonstrated that the unadjusted signal of higher risk observed in those with improved function disappeared when adjusted for baseline function whereas a 1-year deterioration remained prognostic of time to HF hospitalization or death (*Table 3*). In the model adjusted for baseline GLS, GLS improvement was associated with reduced risk when compared with patients with either unchanged or deteriorated function (HR 0.51, CI 95%: 0.29–0.88, *P* = 0.017).

**Table 3:** Cox regression models for direction of change and no change in systolic function with time to first HF hospitalization or all-cause death as dependent variable

Model			Hazard ratio	Confidence interval	*P*-value	Events (n)
LVEF	Unadjusted					
	1	Unchanged	0.50	0.30–0.82	**0.007**	25
	2	Deterioration	3.40	1.47–7.90	**0**.**004**	6
	3	Improvement	1.55	0.94–2.53	0.084	32
	Adjusted					
	1	Unchanged	1.16	0.61–2.21	0.647	25
	2	Deterioration	2.99	1.29–6.97	**0**.**011**	6
	3	Improvement	0.59	0.32–1.10	0.096	32
GLS	Unadjusted					
	1	Unchanged	0.63	0.37–1.10	0.103	18
	2	Deterioration	4.39	2.00–9.64	**<0**.**001**	7
	3	Improvement	1.08	0.65–1.78	0.776	38
	Adjusted					
	1	Unchanged	1.25	0.69–2.26	0.464	18
	2	Deterioration	5.69	2.55–12.70	**<0**.**001**	7
	3	Improvement	0.51	0.29–0.88	**0**.**017**	38

All hazards ratios are reported with the two other categories combined as reference. Adjusted models include baseline LVEF or baseline GLS accordingly to compensate for regression-to-the-mean. Statistically significant *P*-values are reported in bold.

**Figure 4:**
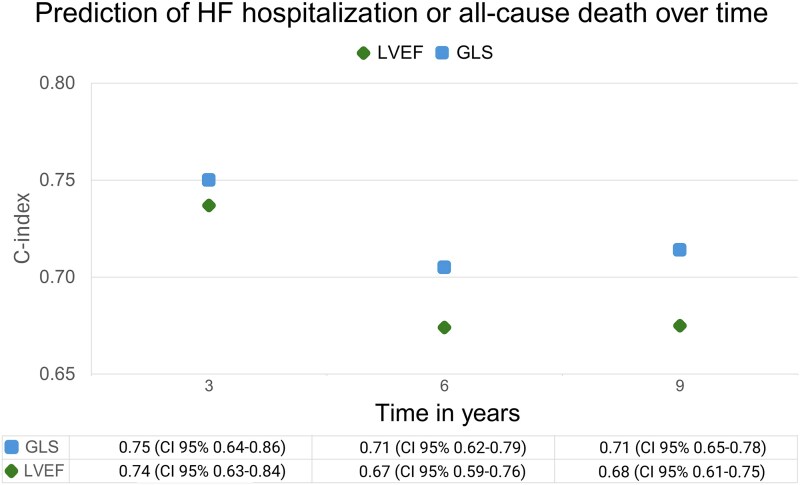
C-index at 3-, 6-, and 9-year follow-up for baseline LVEF and GLS as absolute continuous variables. Description: GLS—global longitudinal strain; LVEF—left ventricular ejection fraction.

In unadjusted Cox regression models, time to first HF hospitalization or all-cause death increased with age, pre-existing diabetes, left atrial (LA) remodelling (increased size and reduced reservoir strain, respectively), higher creatinine and higher C-reactive protein levels, whereas male sex, Peak Troponin I level and type of MI (STEMI vs. non-STEMI) were not associated with the outcome (see Supplementary data online, *Table S5*). LVEF and GLS were associated with the outcome when measured both at baseline and at 1 year from the index hospitalization. Deterioration and improvement in LVEF were both associated with worse outcome when compared to those with no change in this unadjusted model. A deterioration in GLS was associated with worse outcome but improvement was not when compared to no change in GLS. Unadjusted baseline GLS offered a higher C-index than baseline LVEF over time, reflecting a better accuracy in discriminating those with events from those with no events (*Figure 4*).

The multivariable Cox regression model analysing change in LVEF with time to first HF hospitalization or all-cause death as dependent variable (*Table 4*) demonstrate no difference in the outcome between those with unchanged LVEF and those with a 1-year improvement [HR 1.03 (CI 95% 0.51–2.06)]. There were also no differences in outcome between those with reduced LVEF and no change in LVEF. In multivariable model 2 (*Table 4*), evaluating change in GLS, deterioration in GLS was associated with the combined outcome [HR 5.60 (CI 95% 2.04–15.39)] whereas improvement was not [HR 1.05 (CI 95% 0.55–2.04)] when adjusted for baseline age, sex, pre-existing diabetes, creatinine levels, C-reactive protein levels, LA size, and LA reservoir strain. The signal of prognostic value from a 1-year GLS deterioration appeared stable in bootstrapped sensitivity analyses (see Supplementary data online, *Table S6*). Multivariable Cox regression analyses with absolute continuous changes in systolic function demonstrated statistical significance for ΔGLS [HR 0.90 (CI 95% 0.81–0.99) per unit improvement, *P* = 0.033], whereas ΔLVEF did not emerge with statistical significance [HR 1.02 (CI 95% 0.99–1.05) per unit reduction, *P* = 0.221] (see Supplementary data online, *Table S7*).

**Table 4:** Multivariable Cox regression models with time to first HF hospitalization or all-cause death as dependent variable

Model	Variable	Hazard ratio	CI 95%	*P*-value
1	Age, per year increase	1.09	1.06–1.13	**<0.001**
	Baseline LVEF, per unit increase	0.96	0.94–0.99	**0**.**008**
	Diabetes	1.84	0.95–3.57	0.071
	LA size, per ml increase	1.00	0.99–1.01	0.687
	LA reservoir strain, Per unit increase	0.99	0.96–1.02	0.514
	Creatinine, per µmol/L	1.00	0.99–1.01	0.547
	hsCRP, per mg/L	1.00	1.00–1.01	0.446
	Change in LVEF			0.291
	Unchanged	reference		
	Deterioration	2.17	0.77–6.01	
	Improvement	1.03	0.51–2.05	
2	Age, per year increase	1.08	1.05–1.12	**<0**.**001**
	Baseline GLS, per unit increase	1.23	1.12–1.35	**<0**.**001**
	Diabetes	1.85	0.95–3.58	0.069
	LA size, per ml increase	1.01	1.00–1.01	0.257
	LA reservoir strain, Per unit increase	1.00	0.97–1.03	0.969
	Creatinine, per µmol/L	1.00	0.99–1.02	0.427
	hsCRP, per mg/L	1.00	1.00–1.01	0.340
	Change in GLS			**0**.**002**
	Unchanged	reference		
	Deterioration	5.60	2.04–15.39	
	Improvement	1.05	0.55–2.04	

Statistically significant P-values are reported in bold.

## Discussion

In this study, the results did not support the hypothesis that improved systolic function after an MI adds incremental long-term prognostic information beyond the baseline systolic function when compared to patients with no change in function. However, those with a deterioration in GLS over 1 year had a higher risk of events during follow-up compared to those without any change in GLS, although this finding is to be interpreted as exploratory given the small number of patients with GLS deterioration. Furthermore, the results favour a clinical strategy of risk stratification based on baseline systolic function determined by GLS rather than LVEF given its higher long-term C-index.

Patients with improved function demonstrated a slightly higher event rate than those with no change over 1 year in the unadjusted Kaplan–Meier curves; however, these differences were not statistically significant in the Cox regression analyses and the curves only separated after 4 years follow-up. Given that both groups were of similar age, it is plausible that the observed difference in event rate represent late complications from the larger infarction that initially caused lower baseline LVEF in the ‘improved’ population.

A recent study by Caunite *et al.* reported an association between a 1-year deterioration in GLS and mortality within a large cohort which strengthens confidence in our observation.^14^ The change in GLS following MI has previously also been studied by a Danish group that reported no added prognostic value of change in GLS to follow-up GLS alone in assessment of incident heart failure.^15^ A major difference between the Danish study and our study was their lack of definition of a significant change in GLS. It is also worthy to note that GLS change in the mentioned study only lost statistical significance once entered into a model with 13 co-variables, including both baseline and follow-up GLS, thereby suffering from collinearity.^16^

The prediction of time to HF hospitalization or death from 1-year GLS deterioration in essence means that the follow-up acquisition carries independent prognostic information in relation to the baseline acquisition. Complications occurring after discharge, such as reinfarctions, were plausible explanation to a further reduction in systolic function; however, only two hospitalizations occurred within the first year among these patients. The mechanism of continued deterioration in GLS hence remains unknown.

Patients with reduced LVEF at 1-year follow-up had reduced function already at baseline, which would have singled them out as high-risk individuals before discharge, many being eligible for reassessment within 3 months for evaluation of implantable cardioverter defibrillator (ICD) candidacy.^3^ An assessment of GLS deterioration in patients already undergoing this second exam in clinical routine may be a convenient way to identify those at particularly high risk of future complications, should this prognostic signal be confirmed in larger future studies.

Previous positive studies examining improvement in LVEF following MI reported substantially lower overall systolic function from the index exam.^7,17–19^ In the paper by Chew *et al.* looking at data from the ISAR, REFINE and CARISMA cohorts, the overall median baseline LVEF was 40% (IQR 35 to 45). However, only the ISAR cohort included patients also with normal LVEF. In that cohort, baseline LVEF was 42% (IQR 35 to 46) at day 1 post-MI and 48% (40 to 55) at 14 days.^19^ The lower baseline function likely reflect that it is an older cohort (recruited between 1996 and 2005), with patients therefore not fully benefitting from early invasive revascularization. Furthermore, the follow-up LVEF assessments in these three cohorts were all acquired within 6 weeks to 3 months after the index admission and this may have prevented some of these patients from having full effect from their heart failure treatment. The paper by Chew *et al.* stratified patients into no recovery (Δ ≤ 0%), modest recovery (Δ 1% to 9%), and large LVEF recovery (Δ ≥ 10%). This definition of no recovery, including those with a deterioration, will have strengthened power to identify a difference compared to our main analysis defining no recovery as a separate category from deterioration.^20^

The idea of monitoring change in systolic function has proven valuable to guide medical interventions in the realm of cardio-oncology, which encourages evaluation of this approach in additional areas of cardiac disease.^21^ However, management in the MI setting is special. First, baseline LV function is rarely known in advance of the infarction while oncology patients at risk are recommended to undergo a baseline assessment of LVEF in advance of treatment with cardio-toxic medications.^22^ Also, the change in LV-functional metrics following MI mainly reflect a process of recovery in contrast to the deterioration under ongoing exposure to a toxic agent seen in cardio-oncology. Consequently, these disease processes are inherently different. The relative GLS deterioration to identify a cardio toxic effect recommended from the cardio-oncology guidelines is at 15%, which closely resembles the threshold for smallest detectable change used in our study,^22^ strengthening the clinical relevance of the smallest detectable change threshold.

The greatest improvement of systolic function occurs within the first days following an MI.^23^ In a Polish study from 2018 with serial GLS assessments after MI admission, GLS improved from −7.47 ± 3.32 to −14.48 ± 5.50 between Day 1 and Day 3. The Day 3 assessment also best predicted continued improvement of LV-function up until 6 months following admission with an AUC at 0.878 (95% CI: 0.845–0.906) vs. an AUC from the Day 1 exam at 0.718 (95% CI: 0.676–0.758). It is reasonable to assume that the very early improvement within the first days of admission reflects resolved stunning. Given the spontaneous recovery expected within the first days of admission, it appears preferable to delay the baseline exam if clinically feasible. Baseline GLS in our study was −14.3% (± 3.6) which likely reflects that most exams were performed closer to the 72-h mark.

A substantial proportion of events occurred among patients with normal baseline function, highlighting the need for improved risk prediction among patients surviving with preserved systolic function. The presence of high-risk individuals in the sub-population with preserved LVEF is illustrated by the 10-year mortality among MI subjects with LVEF ≥ 50% at 24.8% in a recently published Brazilian study.^24^ GLS has repeatedly been proposed as the solution to the lack of sensitivity often observed in LVEF.^25^ However, this improved sensitivity seems to be marginal in the overall MI population. In a recent comparison of added value from baseline GLS to baseline LVEF after ACS, there was no statistical nor clinical significance in the improvement of Harrell’s C as GLS was entered to the model with LVEF and clinical history.^10^ In our study, GLS deterioration singled out only a few patients who eventually met the endpoint, reflecting a poor sensitivity. However, in the choice between either baseline LVEF or baseline GLS, our findings favour GLS for more reliable risk assessments.

The main reason why MI patients with preserved LVEF do not seem to benefit from additional assessment of their systolic function is likely because their infarct often is too small to be a driving cause of adverse outcome. Instead, it is likely that residual risk among MI patients with preserved LVEF is driven by comorbidities such as renal failure, diabetes, anaemia, peripheral atherosclerotic vascular disease, and chronic lung disease.^26^

### Limitations

This study has limitations. First, there was a substantial number of patients excluded because their exams were not retrievable from the local database (*n* = 95). This was the consequence of a change in echocardiographic data archive and communication system (PACS) provider back in 2019 wherein some of the earliest exams were not automatically transferred to the new database. Another 43 patients were excluded due to degraded image quality. In previous publications on GLS assessments, exclusion due to poor image quality has ranged from 7% to 20%.^27,28^ This wide range is likely due to varying exclusion criteria based on either two or three untraceable segments to prompt exclusion. The 10% excluded based on the two-segment criteria in this present study therefore likely reflects an overall good image quality compared to other cohorts. There is also a selection bias demonstrated by excluded subjects being of older age than included subjects. This difference may in part have been driven by the landmark design with exclusion of patients not surviving until the 1-year follow-up scan, potentially leading to a selection of healthier and younger patients at baseline.

Second, although the sample size was appropriate for detection of associations between the echo measurements and time to first HF hospitalization or all-cause death, there was limited power to perform reliable in-depth sub-analyses.

Third, the use of only one rater is a good way to maximize precision in the assessments of systolic function but prevents any guidance on acquisition accuracy and inter-rater variability although the use of a validated automatic software is likely to have strengthened accuracy in this study.^29,30^ Our core lab has previously reported limits of agreement for inter-rater estimates of LVEF among Swedish MI patients ranging from −16% to 8%, emphasizing the known difficulties in achieving consistent measurements of systolic function by echocardiography.^20^

## Conclusion

Improvement in LVEF or GLS 1 year after MI does not offer incremental prognostic information of time to HF hospitalization or death beyond the baseline assessment alone, whereas a deterioration in GLS may reflect an increased risk of these events, although this needs to be confirmed in a larger and more diverse population. GLS at baseline offers superior discrimination of time to HF hospitalization or death than baseline LVEF and could serve to improve clinical risk prediction.

## Supplementary Material

qyag005_Supplementary_Data

## Data Availability

Researchers are able to access the studied data at Uppsala University upon reasonable request and under the provision that the data is accessed onsite and does not leave Uppsala University. This request can be sent to the corresponding author.

## Lead author biography

Joel Lenell, MD, PhD, is a consultant cardiologist and postdoctoral researcher at Uppsala University Hospital, where he earned his PhD in cardiovascular imaging. After defending his thesis in mid-January 2025, he joined Cedars-Sinai Medical Center in Los Angeles as a postdoctoral scientist focusing on advanced imaging and AI applications in cardiovascular research.
