# Supplementary material for: Long-term prognostic impact of one-year change in left ventricular function after a myocardial infarction: insights from the REBUS cohort
Source: Eur Heart J Imaging Methods Pract. 2026 Jan 12;4(1):qyag005. doi: 10.1093/ehjimp/qyag005 (PMC12835919; doi:10.1093/ehjimp/qyag005)
Supplement: qyag005_Supplementary_Data [file qyag005_supplementary_data.docx]

**Supplemental material**

Inclusion and exclusion criteria:

Inclusion criteria:

- Signed informed consent
- MI diagnosed as a dynamic raise in troponin I with at least one value above the 99^th^ percentile.

Including at least one of the following:

1. Symptoms suggestive of MI
2. Development of a significant Q wave.

Exclusion criteria:

- Death ≤5 days after MI
- Patient living outside the catchment area of Uppsala University Hospital
- Patient being unsuitability for participation in the trial for any reason as judged by the investigator, including inability to attend the scheduled study visits for evaluation procedures.

Supplemental table 1. Comparison between included and excluded patients.

|  | All  (N = 421)^a^ | Included patients  (N = 256) | Excluded patients  (N = 165)^b^ | P-value |
| --- | --- | --- | --- | --- |
| Age, years (SD) | 67 (10) | 66 (10) | 69 (10) | **0.018** |
| Male sex, N (%) | 327 (77.7) | 205 (80.1) | 122 (73.9) | 0.140 |
| STEMI, N (%) | 194 (46.1) | 131 (51.2) | 63 (38.2) | **0.009** |
| Baseline LVEF (%), median (IQR | 55 (47 to 61) | 55 (46 to 61) | 56 (49 to 62) | 0.455 |
| Baseline GLS (%), median (IQR) | -14.7 (-17.0 to -12.3) | -14.7 (-17.0 to -11.9) | -14.7 (-17.2 to -12.8) | 0.809 |

^a^ 352 patients with readable baseline echocardiograms, ^b^ 96 patients with readable baseline echocardiograms

GLS – global longitudinal strain; IQR – interquartile range; LVEF – left ventricular ejection fraction; SD – standard deviation; STEMI – ST-elevation myocardial infarction

Supplemental Table 2. Baseline characteristics among patients with a readable baseline echocardiographic scan not surviving until the follow-up exam.

|  | All  n = 9 |
| --- | --- |
| Male sex, n (%) | 7 (77,8) |
| Age, mean (SD) | 71 (7) |
| Diagnosis of MI |  |
| STEMI, n (%) | 6 (66,7) |
| NSTEMI, n (%) | 3 (33,3) |
| Medical history before MI |  |
| Hypertension, n (%) | 130 (50.8) |
| Diabetes mellitus, n (%) | 2 (22,2) |
| Heart failure, n (%) | 2 (22,2) |
| Chronic kidney disease, n (%) | 1 (11,1) |
| History of MI, n (%) | 5 (55.6) |
| History of stroke, n (%) | 3 (33,3) |
| Blood samples |  |
| Hemoglobin (g/L), median (IQR) | 133 (117-156) |
| Creatinine (umol/L), median (IQR) | 84 (69-122) |
| Peak Troponin I (ng/L), median (IQR) | 10 (2 – 23) |
| C-reactive protein (mg/L), median (IQR) | 9 (4-70) |
| Echocardiography |  |
| LVEF (%), median (IQR) | 52 (39-60) |
| GLS (%), median (IQR) | -11,3 (-16,0 to -9,0) |
| LA volume (ml), median (IQR) | 63 (40-80) |
| LA reservoir strain (%), median (IQR) | 22.8 (15.9-30.5) |

GLS – global longitudinal strain; IQR – interquartile range; LA – left atrium; LVEF – left ventricular ejection fraction; NSTEMI – non-ST-elevation myocardial infarction; MI – myocardial infarction; STEMI - ST-elevation myocardial infarction

|  | 1 vessel disease  (n=174) | 2 vessel disease  (n=55) | 3 vessel disease  (n=26) |
| --- | --- | --- | --- |
| PCI |  |  |  |
| LAD, n (%) | 76 (44) | 30 (55) | 11 (42) |
| LCX, n (%) | 27 (16) | 19 (35) | 11 (42) |
| RCA, n (%) | 54 (31) | 25 (45) | 14 (54) |
| CABG, n (%) | 2 (1) | 0 (0) | 5 (19) |
| Full revascularization, n (%) | 159 (91) | 21 (38) | 8 (31) |
| No revascularization, n (%) | 15 (9) | 2 (4) | 0 (0) |

Supplemental table 3. Extent of obstructive coronary artery disease and treatment strategy

CABG – Coronary artery bypass graft; LAD – left anterior descending artery; LCX – left circumflex artery; PCI – percutaneous coronary intervention; RCA – right coronary artery

Supplemental table 4. Univariable linear regression models with baseline LVEF as dependent variable.

|  | Beta coefficient | CI 95% | P-value |
| --- | --- | --- | --- |
| One-vessel disease, n (%) | 2.64 | -0.31 to 5.59 | 0.080 |
| Two-vessel disease, n (%) | -2.00 | -5.36 to 1.37 | 0.244 |
| Three-vessel disease, n (%) | -2.81 | -7.38 to 1.76 | 0.227 |
| Stenting of LAD, n (%) | -2.31 | -5.07 to 0.46 | 0.102 |
| Stenting of LCx, n (%) | -0.43 | -3.76 to 2.90 | 0.800 |
| Stenting of RCA, n (%) | 0.41 | -2.47 to 3.29 | 0.779 |
| CABG | -4.48 | -10.96 to 6.01 | 0.566 |
| Full revascularization | 1.72 | -1.33 to 4.77 | 0.268 |
| No revascularization | -3.12 | -11.07 to 4.83 | 0.440 |

CABG – Coronary artery bypass graft; LAD – left anterior descending artery; LCX – left circumflex artery; RCA – right coronary artery

Supplemental Table 5: Univariable Cox regression models with time to first HF hospitalization or all-cause death as dependent variable.

| Variable | Hazard ratio | CI 95% | P-value | C-index  (CI 95%) |
| --- | --- | --- | --- | --- |
| Age, per year | 1.10 | 1.07 – 1.13 | **<0.001** | 0.74 (0.68-0.80) |
| Male sex | 1.06 | 0.57 – 1.99 | 0.853 | 0.50 (0.46-0.55) |
| STEMI | 0.89 | 0.54 – 1.45 | 0.635 | 0.51 (0.45-0.57) |
| Hypertension | 1.33 | 0.81 – 2.20 | 0.258 | 0.53 (0.47-0.60) |
| Chronic kidney disease | 1.77 | 0.43 – 7.23 | 0.428 | 0.51 (0.49-0.52) |
| Diabetes | 2.03 | 1.12 – 3.68 | **0.019** | 0.55 (0.50-0.60) |
| RAAS-inhibitors | 1.87 | 0.89 – 3.93 | 0.098 | 0.54 (0.50-0.59) |
| Betablockers | 1.33 | 0.48 – 3.66 | 0.583 | 0.51 (0.48-0.54) |
| Peak Troponin I, per ng/L | 1.00 | 1.00 – 1.01 | 0.996 | 0.47 (0.40-0.55) |
| Hemoglobin, per g/L | 0.98 | 0.97 – 1.00 | 0.068 | 0.57 (0.49-0.64) |
| Creatinine, per umol/L | 1.02 | 1.01 – 1.03 | **<0.001** | 0.59 (0.52-0.66) |
| hsCRP, per mg/L | 1.01 | 1.00 – 1.01 | **0.015** | 0.61 (0.54-0.68) |
| Baseline LA size,  per ml increase | 1.02 | 1.01 – 1.03 | **<0.001** | 0.65 (0.58-0.72) |
| Baseline LA reservoir strain,  per unit increase | 0.95 | 0.93 – 0.97 | **<0.001** | 0.71 (0.64-0.77) |
| Baseline LVEF,  per unit increase | 0.95 | 0.93 – 0.97 | **<0.001** | 0.68 (0.61-0.75) |
| Follow-up LVEF,  per unit increase | 0.94 | 0.92 – 0.96 | **<0.001** | 0.67 (0.60-0.74) |
| Change in LVEF |  |  | **0.002** |  |
| Unchanged | reference |  |  |  |
| Deterioration | 4.56 | 1.87 – 11.14 |  | 0.53 (0.50-0.57) |
| Improvement | 1.82 | 1.08 – 3.01 |  | 0.55 (0.49-0.61) |
| Baseline GLS,  per unit increase | 1.25 | 1.17 – 1.34 | **<0.001** | 0.71 (0.65-0.78) |
| Follow-up GLS,  per unit increase | 1.25 | 1.18 – 1.33 | **<0.001** | 0.74 (0.67-0.80) |
| Change in GLS |  |  | **<0.001** |  |
| Unchanged | reference |  |  |  |
| Deterioration | 5.43 | 2.26 – 13.02 |  | 0.54 (0.51-0.58) |
| Improvement | 1.40 | 0.80 – 2.44 |  | 0.51 (0.45-0.57) |

CI – Confidence Interval; GLS – global longitudinal strain; hsCRP – high sensitivity C-reactive protein; LA – left atrium; LVEF – left ventricular ejection fraction; RAAS – Renin-STEMI – ST-segment elevation myocardial infarction

Supplemental Table 6. Bootstrapped sensitivity analyses by multivariable Cox regression examining stability in GLS deterioration as prognostic marker.

| Model |  | log Hazard Ratio | Confidence interval | P-value |
| --- | --- | --- | --- | --- |
| 1 | GLS deterioration | 1.74 | 1.10 – 2.31 | **<0.001** |
|  | Baseline GLS, per unit increase | 0.24 | 0.17 – 0.31 | **<0.001** |
| 2 | GLS deterioration | 1.34 | 0.55 – 2.59 | **0.002** |
|  | Baseline GLS, per unit increase | 0.22 | 0.14 – 0.31 | **<0.001** |
|  | Age | 0.09 | 0.06 – 0.12 | **<0.001** |
|  | Male sex | 0.19 | -0.48 – 1.02 | 0.552 |
| 3 | GLS deterioration | 1.61 | 0.75 – 2.80 | **0.003** |
|  | Baseline GLS, per unit increase | 0.22 | 0.12 – 0.32 | **<0.001** |
|  | Age, per year increase | 0.08 | 0.05 – 0.12 | **0.002** |
|  | Diabetes | 0.64 | -0.29 – 1.49 | 0.067 |
|  | LA size, per ml increase | 0.01 | -0.00 – 0.01 | 0.113 |
|  | LA reservoir strain, per unit increase | 0.00 | -0.04 – 0.02 | 0.786 |

GLS – global longitudinal strain; LA – left atrium;

Supplemental Table 7. Multivariable Cox regression models with absolute continuous change in LVEF and GLS

| Model | Variable | Hazard ratio | CI 95% | P-value |
| --- | --- | --- | --- | --- |
| 1 | ∆LVEF, per unit reduction | 1.02 | 0.99 – 1.05 | 0.221 |
|  | Age,  per year increase | 1.09 | 1.06 – 1.13 | **<0.001** |
|  | Baseline LVEF,  per unit increase | 0.96 | 0.93 – 0.98 | **0.001** |
|  | Diabetes | 1.89 | 0.97 – 3.67 | 0.061 |
|  | LA size,  per ml increase | 1.00 | 0.99 – 1.01 | 0.699 |
|  | LA reservoir strain,  per unit increase | 0.99 | 0.96 – 1.02 | 0.501 |
| 2 | ∆GLS, per unit reduction | 0.90 | 0.81 – 0.99 | **0.033** |
|  | Age,  per year increase | 1.08 | 1.05 – 1.12 | **<0.001** |
|  | Baseline GLS,  per unit increase | 1.26 | 1.16 – 1.41 | **<0.001** |
|  | Diabetes | 1.67 | 0.86 – 3.23 | 0.128 |
|  | LA size,  per ml increase | 1.00 | 1.00 – 1.01 | 0.367 |
|  | LA reservoir strain, per unit increase | 1.01 | 0.98 – 1.04 | 0.529 |

CI – Confidence Interval; GLS – global longitudinal strain; LA – left atrium; LVEF – left ventricular ejection fraction;

Supplemental Figure 1: Boxplots of LVEF and GLS in patients with events (HF hospitalization or death) and in patients with no events.


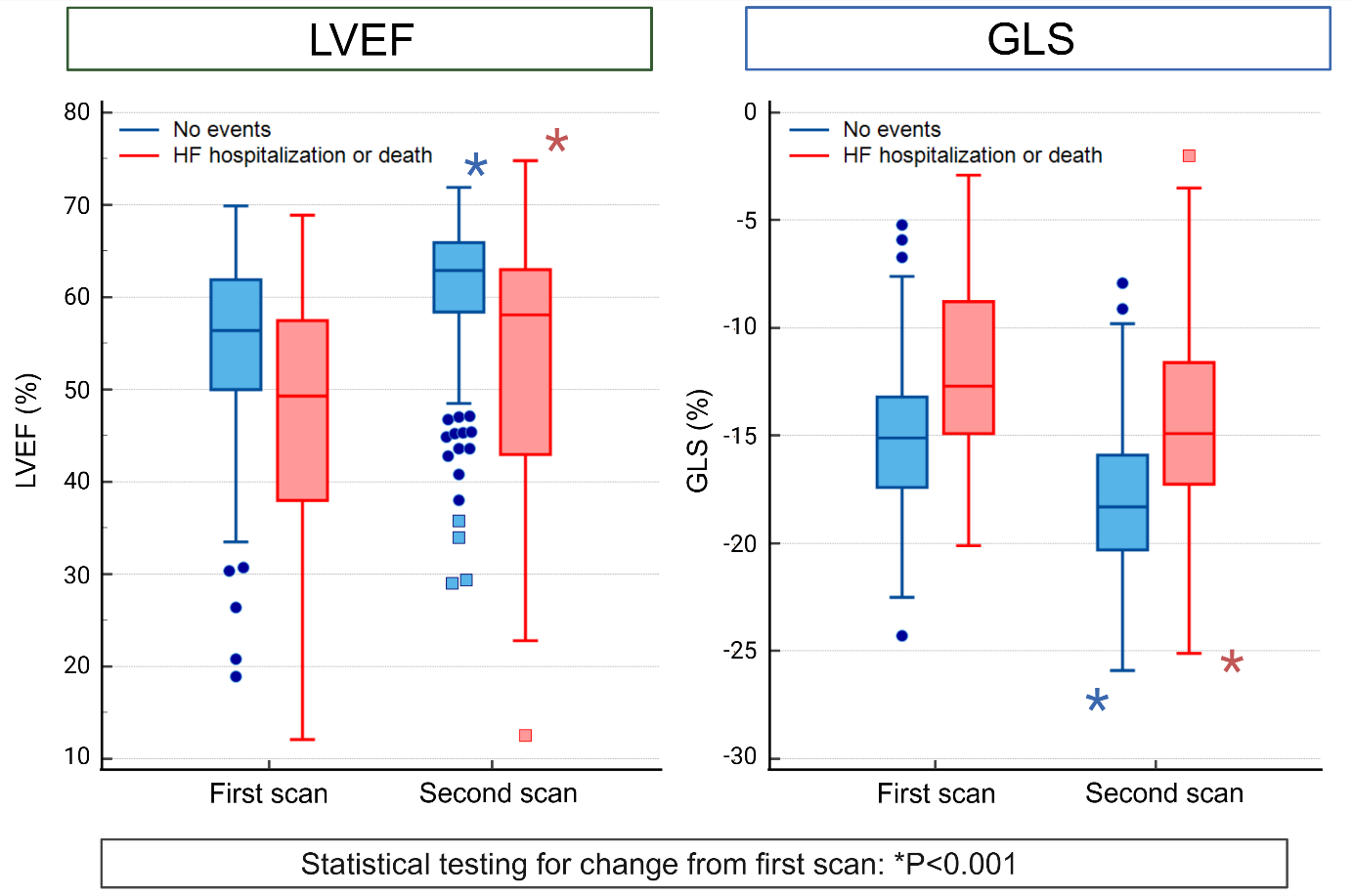


Midline = median; box = inter-quartile range; whiskers = 1.5 x inter-quartile range; first order outliers = >1.5 x inter-quartile range; second order outliers = >3.0 x inter-quartile range.

GLS – global longitudinal strain; LVEF – left ventricular ejection fraction
